# How we do it: artery of Adamkiewicz evaluation by CTA in children

**DOI:** 10.1007/s00247-026-06533-1

**Published:** 2026-02-07

**Authors:** Ankita Chauhan, Ammie M. White, Seth E. Vatsky, Hansel J. Otero, Thomas E. Hamilton, Wondwossen Lerebo, Angel J. Velazquez Guzman, Erfan Akbari, Jordan B. Rapp

**Affiliations:** 1 Department of Radiology, Childrens Hospital of Philadelphia, 3401 Civic Center Blvd, Philadelphia, PA 19104 USA; 2 University of Pennsylvania, Philadelphia, USA; 3 Department of General, Thoracic and Fetal Surgery, Childrens Hospital of Philadelphia, Philadelphia, USA

**Keywords:** Artery of Adamkiewicz, Spinal cord ischemia, Aortopexy

## Abstract

Identifying the artery of Adamkiewicz (AoA) is essential for minimizing the risk of spinal cord ischemia that can result from injury or displacement during aortopexy. A pre-operative CT angiogram (CTA) is commonly requested; however, locating the artery can be challenging due to its small size and variable course. To enhance the visualization of the artery of Adamkiewicz, it is effective to increase the tube current while maintaining a low kV of 70 and raising the Hounsfield unit (HU) trigger threshold. This method adheres to the As Low As Reasonably Achievable (ALARA) principle, ensuring a reliably diagnostic study.

## Introduction

**Fig. 1:**
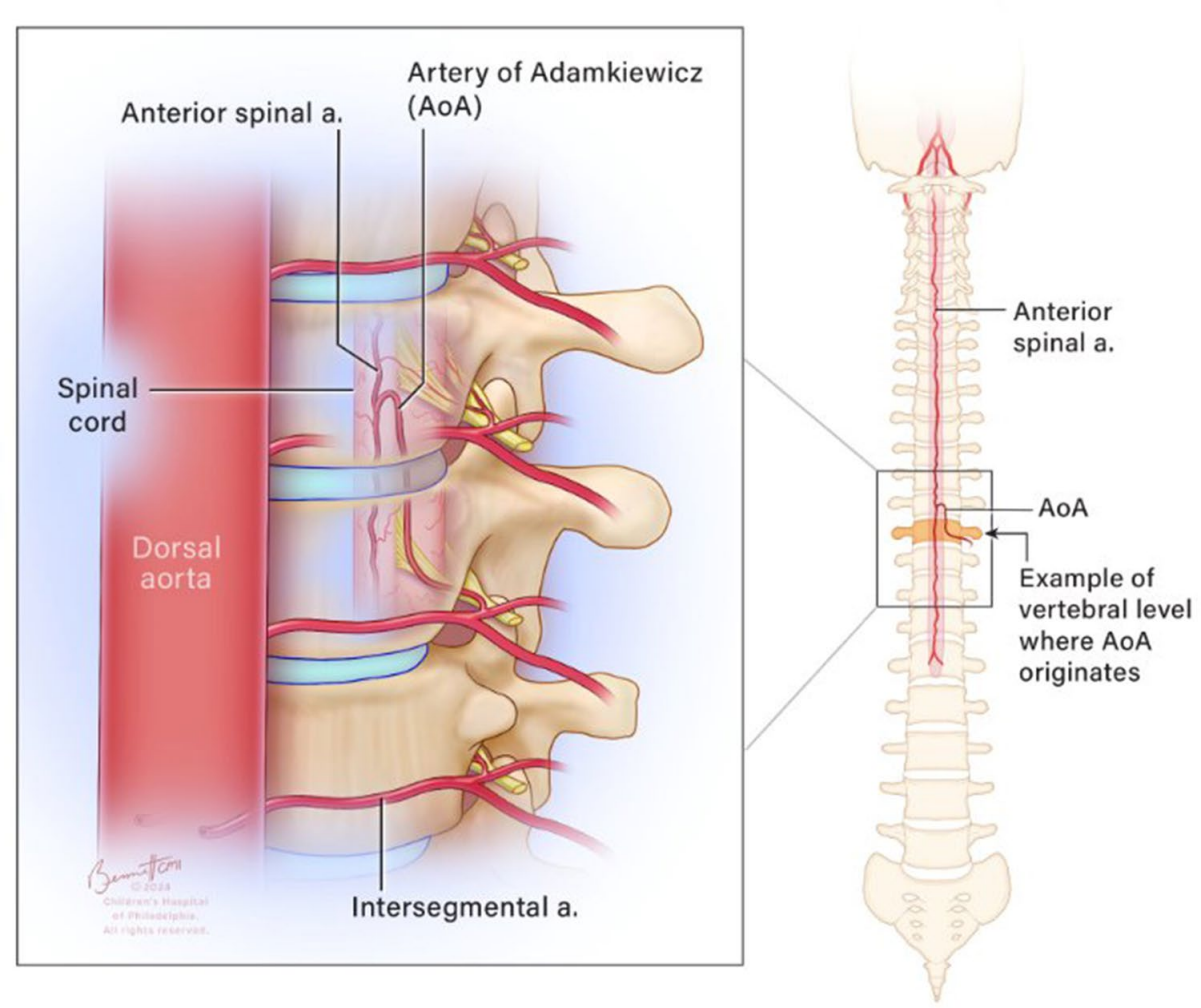
Schematic representation of the artery of Adamkiewicz, arising from a branch of a segmental artery, as it enters via the intervertebral foramen into the spinal canal and takes a hairpin turn before reaching the anterior spinal artery

The artery of Adamkiewicz (AoA), also known as the arteria radicularis magna, is a major vessel that supplies blood to the anterior spinal artery at the lower thoracic and lumbar levels. The AoA is a branch of the radiculomedullary artery, which itself branches off from a segmental artery (either an intercostal or lumbar artery, depending on the vertebral level) that originates directly from the descending aorta (Fig. 1) [1]. Understanding the AoA’s position is crucial before any surgical procedure involving the descending aorta. Injury to the AoA can lead to spinal cord ischemia and may result in paraplegia [2, 3].

## Indication for CTA

**Fig. 2:**
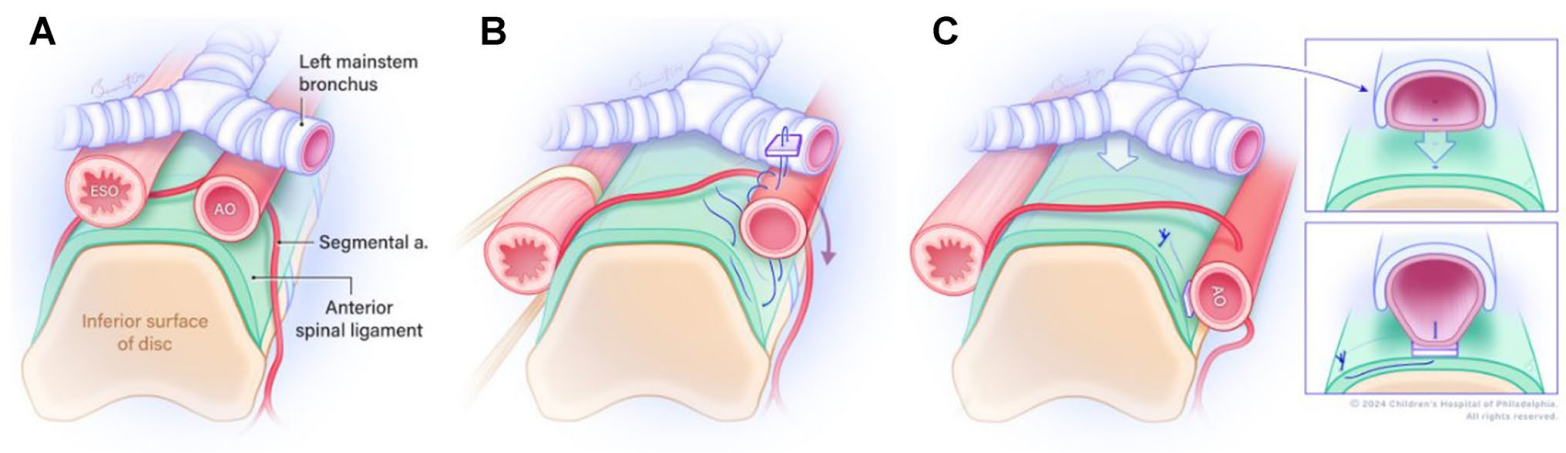
Schematic representation of a posterior descending aortopexy with posterior tracheopexy. **A** Pre-operative view depicts the esophagus (EO) and descending aorta (AO) posterior to the trachea and the mainstem bronchi. **B** Intra-operative view as the EO is mobilized to the right, and the AO is mobilized to the left. Pexy sutures are shown. **C** Post-operative view with close-up panels after completion of aortopexy and posterior tracheopexy depicting the trachea sutured to the anterior spinal ligament

In children, the few indications for AoA assessment include pre-operative assessment before tumor resection [4, 5] and posterolateral descending aortopexy [6]. The latter procedure is routinely performed as an adjunct to posterior tracheopexy in patients with severe tracheobronchomalacia to reduce the pressure exerted by an anteriorly located aorta on the left mainstem bronchus as it transits across the descending aorta (Fig. 2). Posterolateral descending aortopexy may include division of intercostal arteries to mobilize the aorta, making it important to identify the AoA. As a result, select patients diagnosed with tracheobronchomalacia will undergo pre-operative computed tomography angiography (CTA) of the chest to assess the position of the descending aorta in relation to the anterior spinal border and to check for any compression on the left mainstem bronchus. This will help the surgeon decide if posterolateral descending aortopexy needs to be performed in addition to the posterior tracheopexy.

## Rationale behind CTA protocol optimization

Most children who undergo a posterior descending aortopexy are under 5 years of age and usually as young as several months. Historically, accurately and reliably determining the origin of the AoA has been challenging due to its small diameter (0.8–1.3.8.3 mm) and highly variable origin [7]. Standard low-dose CT scans of the chest are inadequate for visualizing a vessel the size of the AoA. Most published literature has primarily focused on adult CTA, magnetic resonance angiography (MRA), or cadaveric evaluations of the AoA [8]. The largest previously published studies in children described 47 patients aged 6 years to 16 years [9] and another with 40 patients aged 5 years to 14 years [10]. A case series focusing exclusively on children included six patients ranging from 6 months to 16 years of age [4]. Other pediatric studies have focused solely on the use of digital subtraction angiography (DSA) [5, 11].

Previous studies have indicated that using a lower tube voltage can enhance the visualization of the AoA [12], and that 64-section CT can improve AoA detection in children compared to 4-section CT [10]. However, there is limited research on additional scan parameters [11] and scan optimization.

Here, we share our experience visualizing the AoA course in pediatric patients using computed tomography angiography (CTA) during surgical planning for tracheopexy and aortopexy, identifying the factors that most significantly enhance AoA visualization.

## How do we obtain CTA for AoA assessment?

At our institution, we perform an ECG-triggered ultra-high-pitch dual-source CT angiography (CTA) using a second-generation dual-source scanner, the Siemens Definition Flash (Siemens Healthcare, Forchheim, Germany).

### Injection setup and rate

We administer a power injection of 2 mL/kg of Omnipaque 350 iodinated contrast (GE Healthcare, Chicago, IL) through the antecubital vein, commonly the right. We avoid lower extremity injections to prevent contamination of the venous plexus, which can limit the depiction of the AoA. The injection rate varies based on the patient’s weight and the size of the peripheral intravenous cannula. For small babies weighing less than 5 kg, we recommend using a 70–80% diluted contrast (mixed with 20–30% saline), followed by a saline flush, injected rapidly as a tight bolus over 5–10 s.

For all others, we administer a 75–90% diluted contrast with a 10–25% dilute tail by mixing a 50/50% contrast-to-saline ratio (versus a 35/65% ratio), followed by a saline flush over a total of 10–15 s.

### Monitoring

The monitoring phase takes place in the proximal descending aorta to align closely with the potential origin of the AoA. An automatic triggering setting is set to 400 Hounsfield units (HU). Although using the left ventricle is a viable alternative, we found that monitoring the descending aorta provided more accurate timing.

### Reference values

**Fig. 3:**
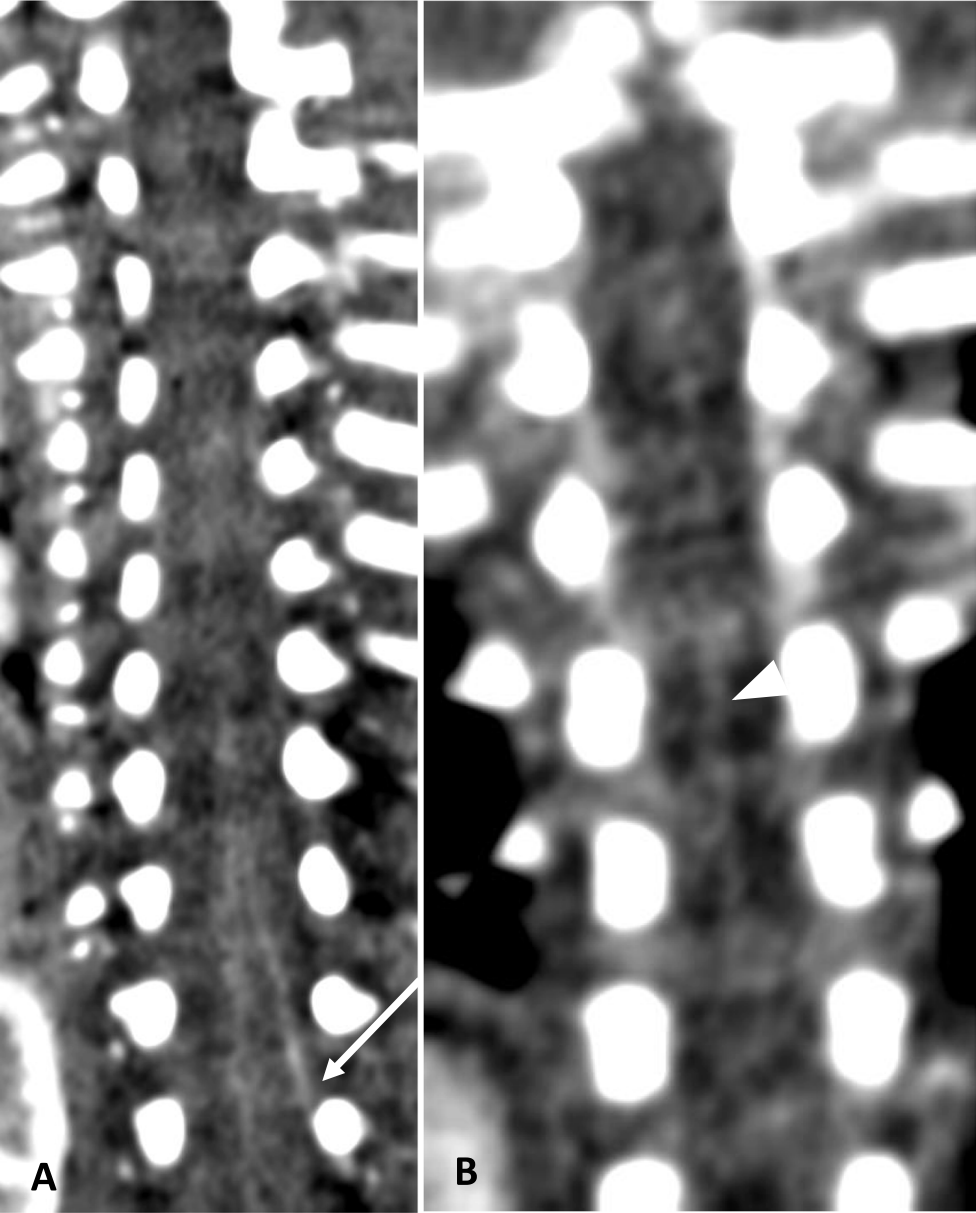
Two patients of similar age. **A** An 11-week-old male with a body surface area of 0.31 m^2^. Coronal CTA chest in soft tissue window. kV=70, mAs=270. The artery of Adamkiewicz can be seen arising from L2 on the left (*arrow*). **B** A 10-week-old female with a body surface area of 0.21 m^2^. Coronal MIP image of the CTA chest in soft tissue window. kV=70, mAs=135. The anterior spinal artery is faintly seen (*arrowhead*), but an artery of Adamkiewicz cannot be identified

We maintained the reference kilovoltage (kV) at 70 to remain close to the k-edge of iodine. This approach improved bolus contrast resolution, making the area of interest easier to identify. To improve the visualization of small vessels, we increased the milliampere-seconds (mAs) setting (Fig. 3). For most patients, we used a reference setting of 350 mAs, but we may raise it to 400–450 mAs for larger patients or those requiring repeat imaging. Table 1 summarizes the CT parameters employed at our institution.

**Table 1:** The CTA parameters utilized in our institution are based on the patient’s body weight

Body weight	kV	Quality ref. mAs	CARE kV	Dose modulation	CTDIvol (mGy) *Based on 300 mAs (effective)	DLP (mGy cm)	Rotation time (s)
Under 20 kg	70	300	On	On	0.81	9.94	0.285
Above 20 kg	70	400	On	On	0.81	9.94	0.285

### Scan range

It is crucial to ensure that all potential origins of the AoA are included within the scan range. Although it has been reported to originate from the cervical region, this is extremely uncommon and lies outside the surgical field. Conversely, while the lower lumbar levels are beyond the surgical area, they are recognized as uncommon origins for the AoA. Including these levels can alleviate concerns about a non-visualized AoA requiring repeat imaging.

The inferior extent of the AoA origin has been documented as low as L4. In our experience, the non-visualization rate was 47% when imaging terminated at L1 or higher, compared with 22% when imaging extended to L4 or lower. Therefore, we recommend including all thoracic levels and lumbar levels up to L4 to ensure completeness.

### A note on radiation

While it is always preferable to image at the lowest possible radiation dose, repeating imaging due to insufficient exposure can result in higher total radiation exposure. The necessity of identifying this vital vessel before surgery—along with the risk of permanent neurological damage should the vessel be injured—justifies a moderate increase in radiation dosage.

### To sedate or not?

With the high-pitch scanning technique, most AoA can be identified without sedation. However, as with any other small structure, sedation can improve visualization. The study can be conducted while the patient is still under sedation from a previous procedure, such as a bronchoscopy or echocardiogram, allowing for a seamless transfer under the same sedation. Additionally, patients who need repeat imaging or are prone to movement may benefit from sedation before the CT scan to avoid excessive radiation exposure from multiple examinations.

### How to decrease artifacts?

In addition to motion-related artifacts, streak artifacts from dense contrast or tubing can also hinder the assessment of the AoA. Diluting the contrast can help reduce the streak artifact. Before starting the contrast injection, always review the CT topogram to ensure there are no high-density enteric tubes or external leads in the field of view. We recommend retracting the enteric tube and repositioning the external tubing before conducting the CTA.

### Which vessel is the artery of Adamkiewicz?

**Fig. 4:**
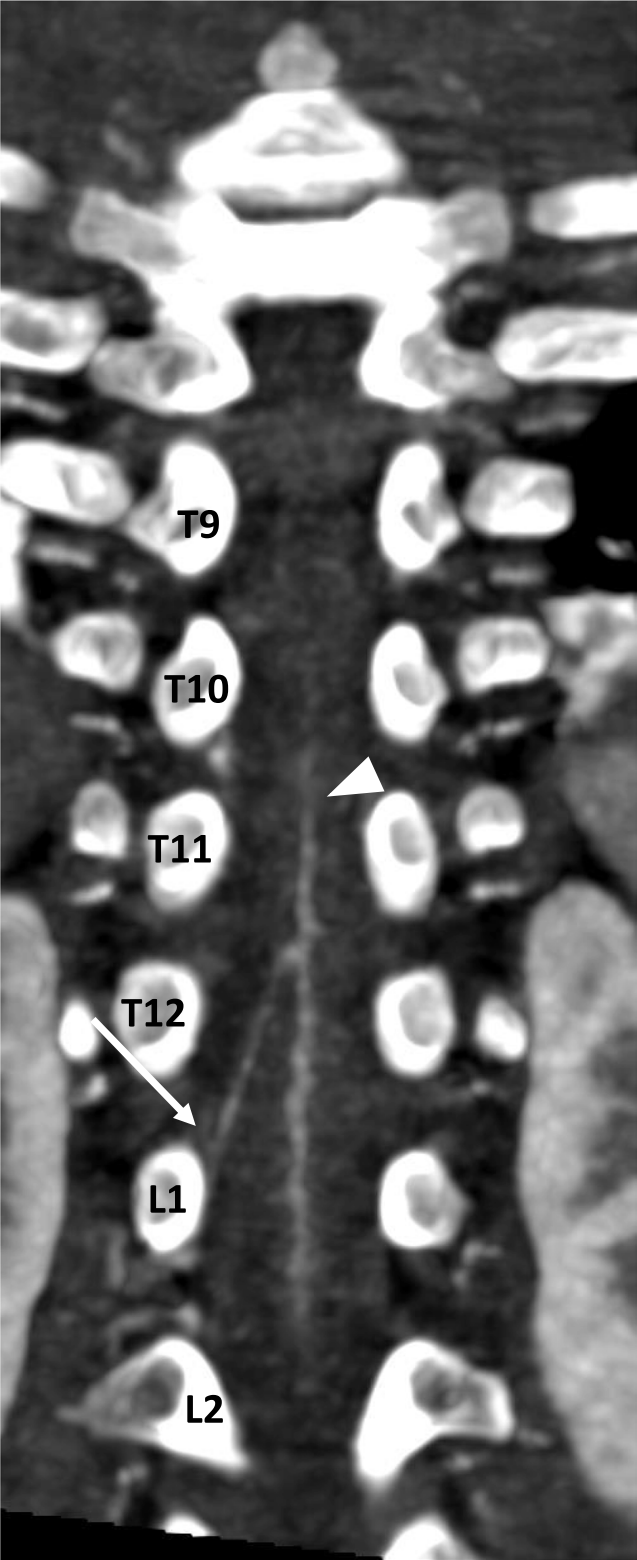
24-month-old male with a body surface area of 0.52 m^2^. Coronal MIP CTA of the chest in soft tissue windows. kV=70, mAs=282. The artery of Adamkiewicz can be seen arising from L1 on the right (*arrow*) feeding the anterior spinal artery (*arrowhead*)

The origin level of the Adamkiewicz artery (AoA) is described as it enters the spinal canal through the intervertebral foramen, specifically at the level of the vertebral body under which it passes (Fig. 4).

**Fig. 5:**
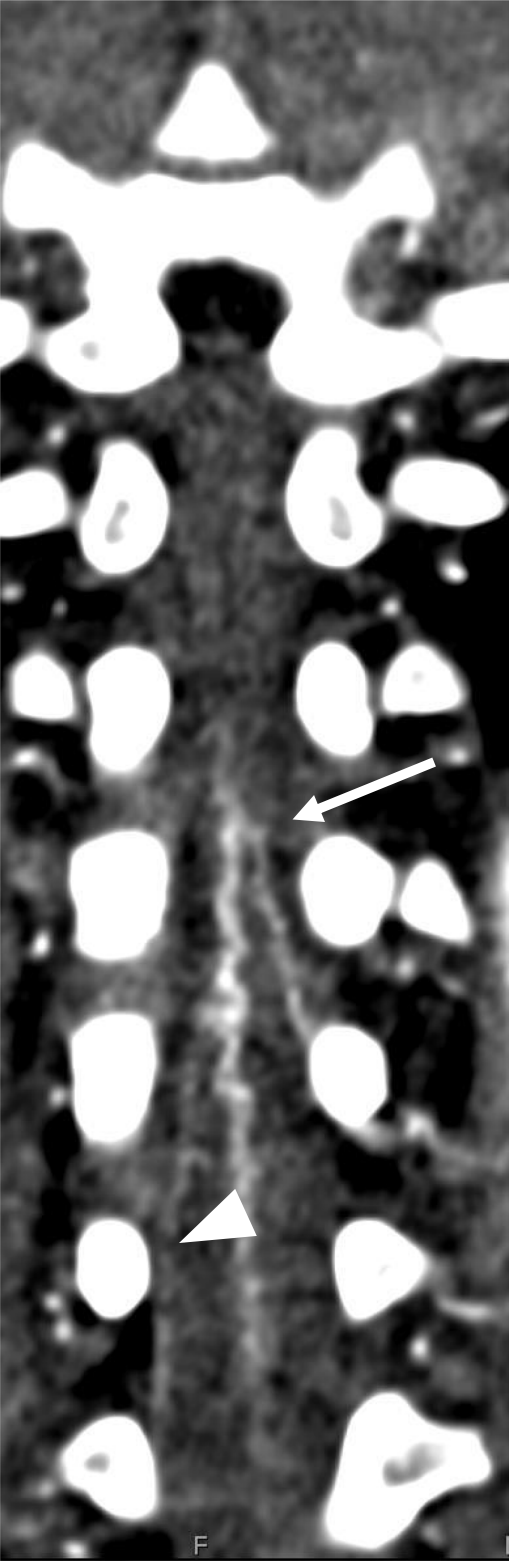
A 23-month-old male with more than one artery feeding the anterior spinal artery. Coronal CTA chest in soft tissue window. The dominant artery on the left (*arrow*) arising from L1 was determined as the artery of Adamkiewicz. The smaller artery on the right (*arrowhead*) was identified as a lesser radicular artery

It is important to note that not all patients possess a distinct AoA. A meta-analysis indicated that up to 15% of patients may lack a defined AoA, with the anterior spinal artery being supplied by multiple intercostal arteries [5]. This same analysis found that 13% of subjects had two or more AoAs, while a study of adult cadavers reported that 26% showed two or more AoAs. Therefore, a single dominant vessel may not be identifiable in a small child. When multiple feeding vessels are present, we consider the largest vessel to be the dominant Adamkiewicz artery (Fig. 5). In the absence of a classic hairpin turn, it can be challenging to distinguish whether a vessel is a true AoA or merely a lesser radicular feeding artery [13].

A meta-analysis involving 5,437 subjects found that the artery is left-sided in 76.6% of cases and located between T8 and L1 in 89% of cases [8]. Reviewing cases from our institution, we observed a left-sided artery in 63% of cases and identified it between T8 and L1 in 74% of instances. This figure increased to 91% when L2 was included in the analysis. Therefore, expanding the scan range to at least L2 will capture most of the AoA; however, we now routinely extend our scans to L3/4 to prevent missing low-origin arteries.

### Post-processing

Minimal post-processing is required. A coronal MIP angled towards the spinal canal may be applicable for visualizing the AoA. The technologist usually performs this at the scanner, but if needed, the radiologist can do it at the workstation.

### Our results

**Fig. 6:**
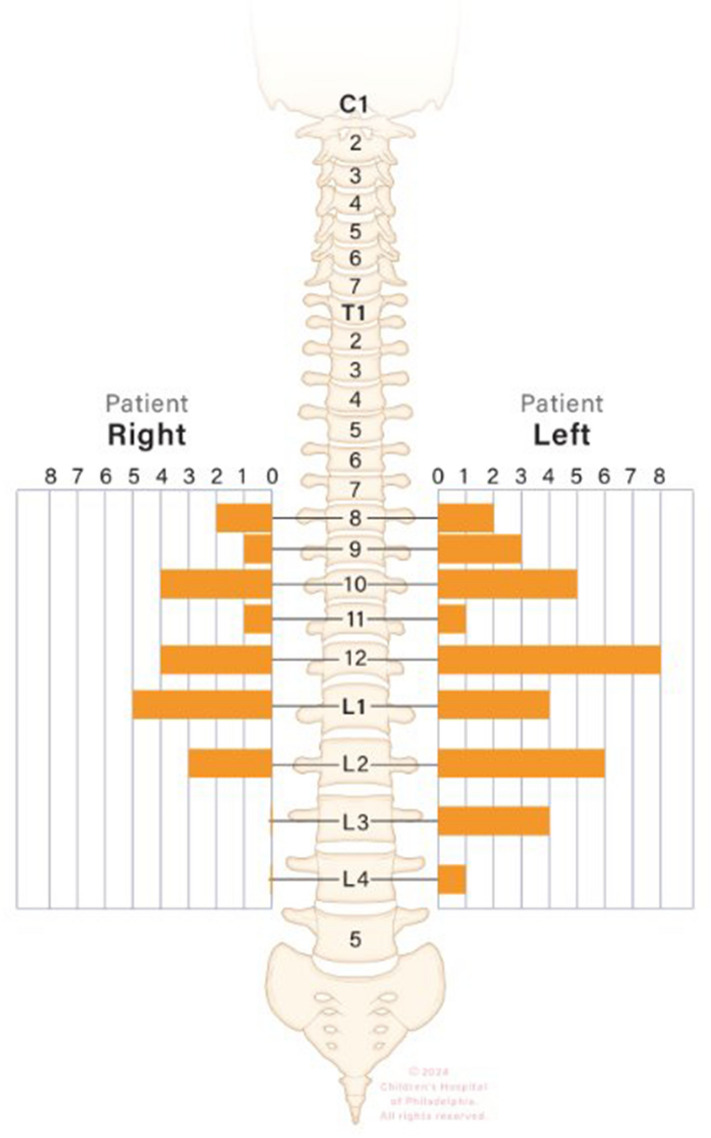
Depiction of the location of each artery of Adamkiewicz origin in our series

At our institution, we have performed 78 examinations on 67 patients (age range 3 days-12 years, median 1.4 years, M/F=37/30) over a period of 20 months. The AoA was visualized in 54/78 (69%). The AoA was seen between T8 and L4 in each case, L/R=34/20. When the scan range terminated at or above L1, the AoA was not visualized in 8/17 (47%); at L2, the AoA was not visualized in 3/8 (38%); at L3, the AoA was not visualized in 9/35 (26%); and at L4 and below, the AoA was not visualized in 4/18 (22%). The AoA was visualized in 20/35 (57%) under 1 year of age, 24/29 (83%) between 1 year and 5 years of age, and 10/14 (72%) between 5 years and 13 years of age. In the visualized group, the median age was 22.1 (6.9–55.8.9.8) months and 5.2 (3.2–30.0.2.0) months in the non-visualized group (*P*-value=0.058), not statistically significant. The median mAs in the non-visualization group=201 and visualization group=281 (*P*=0.005), though no difference was seen by kV (*P*=0.4). Greater Hounsfield units at trigger improved visualization (*P*=0.017). Our findings are summarized in Table 2 and Fig. 6.

**Table 2:** The statistical association between artery of Adamkiewicz (AoA) visualization and background characteristics. *BSA* body surface area, *kV* kilovoltage, *mAs* milliampere-seconds, *CTDIvol* CT dose index measured in milligrays, *TriggerHU* Hounsfield units at trigger location, *DescAoHU* descending aorta Hounsfield units

Characteristics	AoA visualization		
	No	Yes	*P*-value
Sex			
Female	13 (54.2%)	20 (37.0%)	0.158
Male	11 (45.8%)	34 (63.0%)	
Age (months)			
Median (IQR)	5.2 (3.2–30.0.2.0)	22.1 (6.9–55.8.9.8)	0.058
Weight (kg)			
Median (IQR)	5.5 (4.0–12.5.0.5)	11.0 (7.0–17.0.0.0)	0.030
Height (cm)			
Median (IQR)	56.0 (48.0–80.0.0.0)	77.0 (61.0–101.0.0.0)	0.005
BSA (m^2^)			
Median (IQR)	0.3 (0.2–0.6.2.6)	0.5 (0.4–0.7.4.7)	0.015
kV			
Median (IQR)	70.0 (70.0-70.0.0.0)	70.0 (70.0–80.0.0.0)	0.386
mAs			
Median (IQR)	201.0 (112.5–281.5.5.5)	281.0 (236.0–282.0.0.0)	0.005
CTDIvol (mGy)			
Median (IQR)	0.6 (0.4–0.8.4.8)	0.8 (0.8-0.8.8.8)	<0.001
TriggerHU			
Median (IQR)	366.5 (225.0–450.0.0.0)	429.0 (385.0–494.0.0.0)	0.017
DescAoHU			
Median (IQR)	760.0 (565.5–940.5.5.5)	837.5 (604.0-1.0,018.0)	0.258

## Discussion

Published studies on AoA identification are limited to small samples of children and older patients [14]. While studies have shown that CT can identify the AoA, the significance of tube current parameters was not assessed [10, 15]. The optimal scan parameters for identifying the AoA involve increasing the tube current while keeping the kV setting low at 70 and setting a higher HU trigger threshold of 400 or higher.

We strive to maintain a low kV during each exam, regardless of the patient’s size. This approach is crucial not only for imaging smaller patients but also for approaching the iodine k-edge as closely as possible, given scanner limitations. Consequently, it is not surprising that one study in adults found that a lower 100-kV protocol enhanced visualization of the AoA compared with a higher 120-kV protocol [12]. The benefits of increasing the tube current in imaging have not been thoroughly investigated [8]. However, similar improvements have been noted in the evaluation of other small vascular structures, such as the coronary arteries [16]. It has been observed that higher tube current improves visualization of the artery of Adamkiewicz. Therefore, we recommend setting the kV to 70 and using a mAs range of 300–350 to improve the likelihood of identifying the AoA before surgery. It is important to note that there is only marginal improvement in detectability at even higher tube currents.

Breath-holding may not be feasible because many patients are too young or not sedated, and further research is necessary to explore whether sedation, especially in small patients, could be beneficial in complex cases. A potential challenge when planning a scan is that many patients referred for aortopexy may have rib or vertebral anomalies associated with syndromes such as VACTERL, which could complicate rib counting for AoA origin localization, or for estimating scan range. Finally, as imaging techniques and CT hardware and software algorithms improve, particularly with the enhanced spatial resolution provided by photon-counting detectors, identifying the AoA should become easier [17].

## Conclusion

We shared our experience with the artery of Adamkiewicz visualization using computed tomography angiography in children as young as a few weeks old. We recommend increasing the tube current while keeping the kV low and raising the Hounsfield unit trigger threshold. This approach improves the identification of the artery of Adamkiewicz in children, regardless of their age. This technique adheres to the As Low As Reasonably Achievable (ALARA) principle and produces consistently diagnostic results.

## Data Availability

No datasets were generated or analysed during the current study.
